# Predictors, Neuroimaging Characteristics and Long-Term Outcome of Severe European Tick-Borne Encephalitis: A Prospective Cohort Study

**DOI:** 10.1371/journal.pone.0154143

**Published:** 2016-04-25

**Authors:** Thorsten Lenhard, Daniela Ott, Nurith J. Jakob, Mirko Pham, Philipp Bäumer, Francisco Martinez-Torres, Uta Meyding-Lamadé

**Affiliations:** 1 Neuroinfectious Diseases Group, Department of Neurology, University Hospital of Heidelberg, Heidelberg, Germany; 2 Department of Neuroradiology, University Hospital of Heidelberg, Heidelberg, Germany; 3 Department of Neurology, Krankenhaus Nordwest Frankfurt, Frankfurt, Germany; Washington University, UNITED STATES

## Abstract

**Background and Objectives:**

Tick-borne encephalitis (TBE) still represents a considerable medical and health economic problem in Europe and entails a potential threat to travellers. The aim of this study was to characterise the conditions of severe TBE by precisely recording its clinical variants, the related neuroimaging features, and the variant-specific long-term outcome and by identifying predictors for severe courses.

**Methods:**

A cohort of 111 TBE patients (median age 51, range 17–75 years; 42% females) was analysed prospectively. Data were acquired from the department of neurology, University Hospital Heidelberg, and the infectious diseases registry of the Robert-Koch institute Berlin. Neurological status was ascertained by protocol at admission and discharge and the degree of disability was scored using the modified RANKIN Scale (mRS; clinical score addressing neurological disability, range from 0, healthy to 6, dead) at admission and at follow-up. Follow-up examination was conducted by means of a telephone interview. To identify independent predictors for severe TBE and functional outcome, modelled logistic regression was performed. MRI changes were correlated with infection variants. To assess alpha-motor neuron injury patterns, we used high-resolution magnetic resonance neurography (hrMRN). Analyses were performed at the Department of Neurology, University Hospital, University of Heidelberg from April 2004 through September 2014

**Results:**

Acute course: 3.6% of patients died during the acute infection. All patients with a lethal course suffered from meningoencephaloradiculitis (MER, 14.4% of the cohort), which is associated with a significantly higher risk of requiring intensive care (p = 0.004) and mechanical ventilation (p<0.001) than menigoencephalitis (ME, 27.9% of the cohort). At admission, both MER and ME groups were severely affected, with the MER group having a statistically higher mRS score (median of 5 in the MER groups versus 4 in the ME group; p<0.001). Long-term outcome: outcome for MER was considerably worse (median mRS = 4) than for ME (mRS = 1, p<0.0001) and meningitis (mRS = 0, 57.7% of the cohort). Risk factors: advanced age (p<0.001) and male gender (p = 0.043) are independent risk factors for a severe infection course. Furthermore, we identified pre-existing diabetes mellitus (p = 0.024) as an independent risk factor for MER. In MER, alpha-motor neuron injury accounts for the poor prognosis confirmed by hrMRN.

**Conclusion and Relevance:**

These data provide critical information for neurologists and other health professionals to use in evaluating TBEV patients who live in or travel to endemic areas. This information can be used to classify clinical presentation and estimate infection-associated complications and individual prognosis. Furthermore, the risk for severe, disabling infections in older patients should prompt general practitioners to recommend and encourage vaccination to those patients living in or travelling to endemic areas.

## Introduction

Central European encephalitis is the European representative of a group of encephalitides caused by TBEV (tick-borne encephalitis virus). Based on comparative sequence analysis, three related TBEV subtypes causing meningoencephalitis along the 7°C isotherm in Eurasia can be distinguished [1, 2]. The European subtype extends north-east to the Baltic states, east to Ukraine, south-east as far as former Yugoslavia and north to the Baltic Sea coast of Sweden. The Rhine valley in Germany marks the western boundary [2, 3]. TBEV is an enveloped, positive-stranded RNA virus (40–60 nm) belonging to the *Flaviviridae*, a family embracing several other relevant human pathogens, such as Dengue, Japanese encephalitis, Yellow fever and West Nile virus[4, 5]. The main vector in Europe is the tick *Ixodes ricinus*, which, besides TBEV, transmits a range of other pathogens [6]. The incubation period for TBEV is approximately 7–14 (occasionally up to 28) days. Symptomatic infection occurs in approximately 30% of infected patients. It then follows a typical biphasic course with a flu-like prodrome, representing viremia, and CNS infection in approximately 10% of cases.

In endemic regions in central Europe, TBE is the most common viral meningoencephalitis, with incidences far higher than all other sporadic viruses [7–10]. Though a preventative vaccine is widely available, vaccination rates are low in at-risk populations. As a consequence, the annual infection rate is still high in endemic regions (up to 29/100.000) and incidence of TBE has increased since the mid-1990s in most European countries [3, 7]. In most cases, patients suffer from pure meningitis (~ 50%) with overall good prognoses to recover completely. The other half suffers an infection of the brain parenchyma, resulting in meningoencephalitis (~35%), or very rarely an isolated infection of the spinal cord (less than 1%); 1–2% of patients will die from their infection. Approximately 10% of patients will develop infection of the motor neurons (radiculitis) in addition to meningoencephalitis [11]. The clinical presentations of TBE has been categorised according to the focus of the infection into pure meningitis, meningoencephalitis (ME) and menigoencephaloradiculitis (MER). However, a precise classification of clinical presentations as well as associated neuroimaging findings is lacking in the literature. Furthermore, it is not exactly known what patient-related factors are associated with severe infection and poor outcome. Thus, we analysed a cohort of 111 TBE patients from the Odenwald hills region, a high-risk endemic area in south-western Germany, and focused on precise analyses of the acute infection course and the related long-term outcome of the different clinical presentations and aimed to identify risk factors for severe infection with poor outcome. Furthermore, we investigated the related neuroimaging features using not only conventional MRI but also high-resolution magnetic resonance neurography (hrMRN) to cover peripheral motor-neuron injury.

## Patients and Methods

### TBE data acquisition and case definition

TBE represents a certifiable infection in Germany and, by law, must be reported. Data were acquired from our departmental databases, from the district public health offices of the endemic region of the Odenwald hills and from the infectious diseases registry of the RKI Berlin. The study was approved by the ethics committee of the University of Heidelberg and written informed consent was obtained from participants. The data were collected mainly prospectively and in part retrospectively from April 2004 through September 2014.

TBE was identified using a common clinical definition of meningitis confirmed by the presence of anti-TBEV IgM antibodies in the serum and CSF as well as pleocytosis in the CSF. Clinical TBE presentations [11] were defined as follows: “pure meningitis” (meningitis), without focal neurological symptoms; meningoencephalitis (ME), meningitis plus focal neurological symptoms and/or disturbance of consciousness and/or higher-order brain dysfunction; or meningoencephaloradiculitis (MER), ME plus clinical signs of alpha-motor neuron injury (AMNI). All patients were examined at admission and followed up after 12 months at the earliest with a clinical assessment and by recording modified RANKIN scale (mRS)[12]. The mRS is an established clinical score to ascertain the grade of neurological disability and a patient’s ability to perform daily activities and ranges from 0, healthy/no symptoms to 6, dead (1, no significant disability, 2, slight disability, 3, moderate disability, 4, moderate severe disability, 5, severe disability). If recovery was good at discharge (mRS<2), and in all meningitis patients, a follow-up examination was conducted by means of a telephone interview (for items, see Table 1). In cases of clinical evidence of pre-existing polyneuropathy, TBE-related motor-neuron injury was verified by electroneuro- and myography.

**Table 1:** Baseline characteristics of the TBE cohort.

	All patients	MER	ME	Meningitis	
Parameters	n = 111	n = 16 (14.4%)	n = 31 (27.9%)	n = 64 (57.7%)	P-value
Age, median^†^	51 (17–75)	62 (50–75)	57 (17–74)	46 (17–70)	<0.001* 0.009^#^
Female^‡^	47 (42.3%)	3 (18.8%)	9 (29.0%)	35 (54.7%)	0.01* 0.027^#^
Biphasic course^‡^	47 (42.3%)	3 (18.8%)	14 (45.2%)	30 (46.9%)	0.049*
Tick bite^‡,^^§^	71 (72%)	7 (53%)	21 (67%)	43 (79%)	0.07*

Statistic was calculated with Mann-Whitney *U*-test^†^ and with Fisher exact test^‡^, respectively. P-values assigned as follows: MER* and ME^#^ compared to meningitis.

^§^Information was not available in all patients.

CSF was analysed at admission and included complete cell count and anti-TBEV-IgM+IgG antibody detection (serum/CSF) (IMMUNOZYM FSME^®^ Progen, Heidelberg, Germany). In initially uncertain cases, especially patients with a history of vaccination, follow-up serological examination and lumbar puncture had to confirm TBE either by an increase in anti-TBEV-IgG in serum, proof of anti-TBEV-IgM in the CSF or proof of a positive antibody CSF/serum index (anti-TBEV IgG).

### Neuroimaging

For conventional brain MRI (T1,2-weighted-, diffusion weighted -, Flair imaging), see respective textbooks of radiology [13]. In clinically suspected MER, hrMRN was performed to localise AMNI. The spinal nerves and brachial plexus were examined with a two-element multi-channel surface array coil and peripheral nerves with an eight-channel phased-array extremity coil (NORAS GmbH, Höchberg, Germany). HrMRN sequences included 2D fat-saturated T2-weighted sequences in perpendicular orientation to roots, brachial plexus and peripheral nerves[14, 15].

### Statistics

Statistical analyses were performed using the SPSS® software (Version 21). Data were analysed by Fisher’s exact test (nominally scaled) or the Mann–Whitney *U*-test (ordinally scaled). Differences with a probability of p < 0.05 were defined as significant. To identify independent predictors for severe TBE and functional outcome, a binary logistic regression model was performed for all items except for age, where an ordered logistic regression with the Stata® Data Analysis was conducted (Version 12, StataCorp LP, Texas, U.S.A.).

## Results

### Baseline characteristics

The clinical TBE variants were distributed as follows: MER (n = 16, 14.4%), ME (n = 31, 27.9%) and pure meningitis (n = 64, 57.6%). The patients’ median age differed significantly: MER (62 years; p = 0.001), ME (57 years; p = 0.009) and meningitis (46 years). The MER group (n = 3 females, 18.8%, p = 0.01) and the ME group (n = 9 females, 29%, p = 0.027) included significantly fewer females than the meningitis group (n = 35 females, 54.7%). The mean for pleocytosis was 124.3 cells/μL ± 12.2 standard error of mean (SEM). Detailed baseline characteristics are summarised in Table 1.

### Clinical characteristics and long-term outcome in TBE

**Fig 1:**
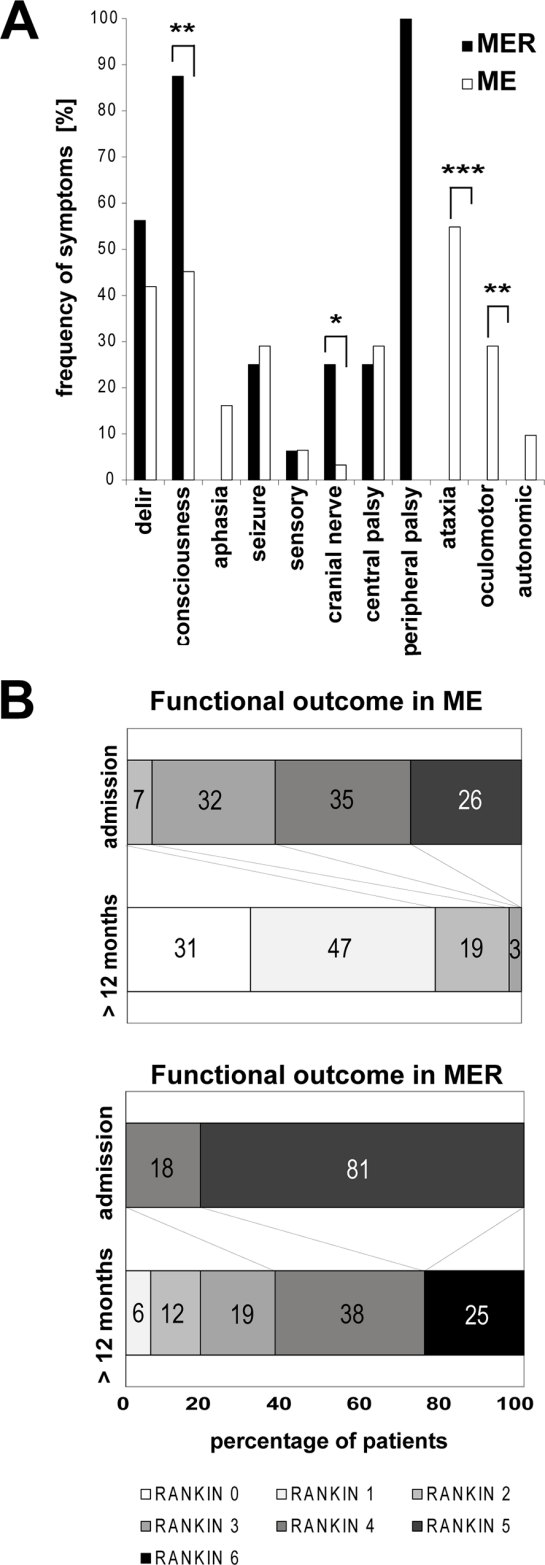
Spectrum of neurological impairment in severe TBE. Frequency of neurological symptoms during acute neuroinfection divided into complex (first four items) and focal neurological symptoms (following 7 items) in MER and ME, respectively (A). Note that peripheral palsy occurred only in MER and not in ME, owing to the clinical definition of these TBE variants. Significance is depicted as p < 0.05 (_*_), p < 0.01 (_**_), and p < 0.001 (_***_), calculated with Fisher’s exact test. Functional long-term outcome in severe TBE (B): Functional outcome of MER and ME according to modified RANKIN scale (mRS) [12] at admission compared with long-term outcome as shift analysis by degree of disability. Numbers within the bars are given in percent (rounded). MRS scale: 0, no symptoms at all; 1, no significant disability, able to carry out all usual activities, despite some symptoms; 2, slightly disabled, able to look after own affairs without assistance, but unable to carry out all previous activities; 3, moderate disability, requires some help, but able to walk unassisted; 4, moderately severe disability, unable to attend to own bodily needs without assistance; 5, severe disability, requires constant nursing care and attention, bedridden, incontinent; and 6, dead.

The spectrum of clinical symptoms at admission is illustrated in Fig 1A. Among the complex symptoms, disturbance in consciousness was observed significantly more often in MER patients (n = 14, 87.5%, p = 0.003) than in the ME group (n = 14, 45.2%). Among the focal neurological symptoms, more MER patients (25%) suffered from cranial nerve paresis than ME patients (n = 1, 3.2%, p = 0.04). ME patients presented with brainstem and cerebellar symptoms significantly more frequently (ataxia: ME group, n = 17, 54.8% versus MER group, n = 0, p = 0.0002; oculomotor disturbance: ME, n = 9, 29% versus n = 0, p = 0.018). Although statistically not significant, it is interesting to note that aphasia (n = 5, 16%) and autonomic disturbances (n = 3, 9%) were observed exclusively in the ME group.

At admission, the MER patients were significantly more severely affected than the ME patients (median mRS 5, versus median mRS 4, p < 0.001) (Table 1). Significantly more MER patients required intensive care (87.5% versus 41.9%, p = 0.004) and more needed to be mechanically ventilated (50% versus 3.2%, p = 0.0002) than ME patients (Table 2).

**Table 2:** Clinical characteristics of severe TBE.

	MER	ME	
Parameters	n = 16	n = 34	P-value
**Disability**^†^mRS, median (min.-max)			
admission^†^	5 (4–5)	4 (2–5)	<0.001
> 12 month^†^	4 (1–6)	1 (0–3)	<0.0001
**Intensive Care**^‡^ n (%)	14 (87.5%)	13 (38.2%)	0.004
Mechanical ventilation^‡^	8 (50%)	1 (2.9%)	<0.001
Tracheotomy^‡^	3 (18.8%)	1 (2.9%)	n.s.
**Co-morbidity** n (%)			
Diabetes mellitus^‡^	7 (43.8%)	4 (11.8%)	0.024
Polyneuropathy^‡^	4 (25.0%)	0 (0.0%)	0.008
Alcohol consumption^‡^	5 (31.2%)	5 (14.7%)	n.s.
others^‡^	3 (18.8%)	3 (8.8%)	n.s.
**Neuroimaging** n (%)			
Pathological MRI^#^ findings^‡^	7 (58.3%)	4 (17.4%)	0.016

MER, meningoencephaloradiculitis; ME, meningoendephalitis; mRS, modified RANKIN scale [12].

Statistic was calculated with Mann-Whitney U-test† and with Fisher exact test‡, respectively.

#MRI was not available in all patients.

As for the long-term outcome, significantly more MER patients were dependent on others in their daily activities, with a high median mRS of 4 (range 1–6) compared with a low median mRS of 1 in the ME group (range 0–3, p < 0.0001) (Table 2). A shift analysis of disability in MER and ME allows a closer look at the degree of disability (Fig 1B): nearly all of the ME patients achieved a good outcome of no or mild disability (mRS 0–2)–except 1 (mRS 3), whereas only 18.8% of MER patients were in this category. Significantly more MER patients (56.3%, p < 0.001) still suffered from moderate or severe disability (mRS 3–5). MER alone accounted for the high mortality (Fig 1B).

### Predictors for severe course and worse outcome

Higher age was observed significantly more frequently in MER (62 years, p = 0.001) and ME (57 years, p = 0.009) than in meningitis (46 years) (Table 1). Using ordered logistic regression analysis to prove independency from co-variables, age is an independent risk factor for developing ME and MER (p < 0.001, odds ratio 1.05, 95% CI 1.02–1.09) (Table 3). This means that every additional year (ten years) of age results in a 5% (50%) higher risk to switch from category meningitis to ME and from ME to MER, respectively. Furthermore, male gender is found more frequently in MER (81.2%, p = 0.01) and ME (71%, p = 0.027) than in meningitis (43.3%). Binary logistic regression of gender predicting outcome showed that men, with an odds ratio of 2.41 (95% CI: 1.03–5.63), were at a significantly increased risk (p = 0.043) of unfavourable outcome of MER (Table 3). In searching for further clinical risk factors and possible predictors for MER, we identified pre-existing DM (p = 0.024) and PNP (p = 0.008) in the MER group. Using binary logistic regression analysis, DM remained an independent risk factor for MER (p<0.024, odds ratio 5.25, 95% CI: 1.24–22.19) (Table 3). Regression analysis was not feasible for polyneuropathy (PNP) since PNP most likely was a dependent variable of DM as a secondary, long-term complication. MER tended to take a monophasic course.

**Table 3:** Predictors for severe TBE.

Dependent variables: TBE variants			
Parameters included	P-value	OR	95% CI
Age^§^	< 0.001	1.05	1.02–1.09
Male^$^	0.043	2.41	1.03–5.63
Diabetes mellitus^$^	0.024	5.25	1.24–22.19
Monophasic-course^$^	0.063	2.31	0.96–5.59

OR, odds ratio; CI, confidence interval

^§^Ordered logistic regression for age: 5% risk per every additional year of age to develop a severe course (meningitis → ME → MER)

^$^Binary logistic regression: gender (meningitis/ME → MER), diabetes and infection course (ME → MER).

### Neuroimaging in TBE

**Fig 2:**
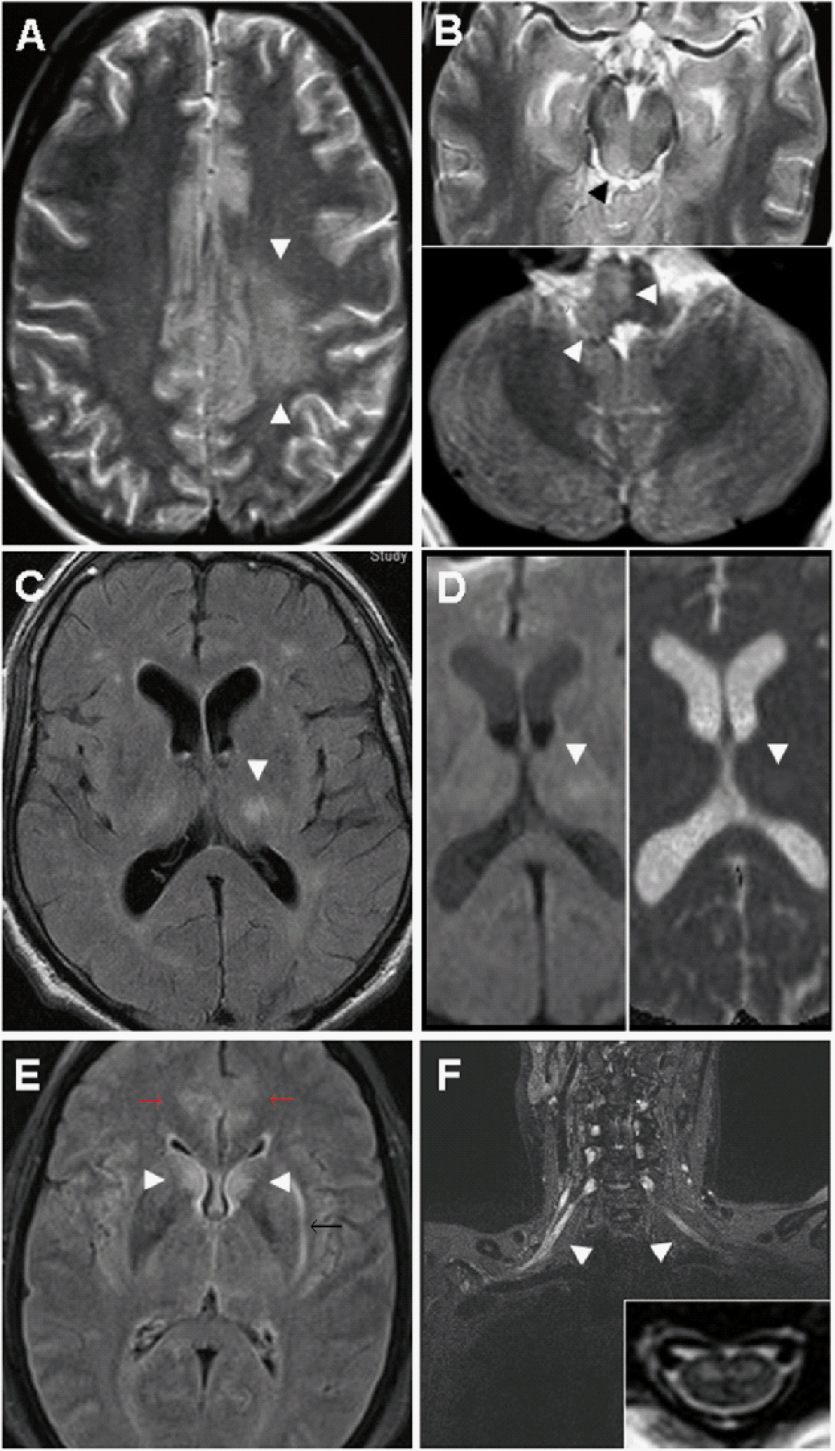
Neuroimaging findings in severe TBE. (A and B), A 23-year-old male patient with severe ME and extensive panencephalitis but complete recovery after 12 months. MRI at admission showed T2-weighted axial lesions of the left semioval centre (A, arrowhead), right inferior colliculum tecti (B, upper panel) and right medulla oblongata (B, lower panel) Not shown: additional T2-weighted lesions in the thalamus and cerebellar peduncles. (C and D), A 62-year-old male MER patient initially misdiagnosed as lacunar stroke with consciousness disturbance, complete hemiplegia, dysphagia and lethal course due to aspiration pneumonia: axial fluid-attenuated inversion recovery (FLAIR) sequence depicts a rounded hyperintense lesion in the left thalamus (arrowhead) (C) that shows pathological water diffusion on diffusion-weighted imaging (DWI) (D, upper panel). Corresponding ADC map identifies the lesion as vasogenic oedema with enhanced water diffusion (D, lower panel), incompatible with acute or subacute stroke. (E and F), A 49-year-old female MER patient with “man in the barrel syndrome” (unable to move her upper limbs) as long-term outcome. MRI at admission showed extended encephalitis, cervical myelitis and brachial radiculitis: axial FLAIR image depicts bilateral hyperintense lesions of caudate nucleus (arrowheads), cingulate cortex (red arrows), and left external capsule (black arrow) (E). A T1-weighted, fat-saturated coronal image of the brachial plexus shows bilateral contrast enhancement of the displayed nerve roots and fascicles, proving radiculitis (F). Inset: A T2-weighted axial image of the cervical myelon shows bilateral, symmetrical, hyperintense lesions strictly confined to the anterior horn.

Among patients with severe infections, significantly more MRI abnormalities of any kind were seen in MER (n = 7 of 12, 58.3%, p = 0.0047) than in ME (4 of 23, 17.4%) (Table 2). Differentiated further into central brain lesions and peripheral lesions, respectively, brain lesions were present in both MER (n = 3 of 12, 25%) and ME (4 of 23, 17.4%) and did not differ significantly (p = 0.67). The thalamus was involved in all patients presenting with cerebral lesions and in two of them, additional lesions could be detected as depicted in Fig 2A–2F. Note that MRI was not available in all patients. HrMRN was not established before 2010.

**Fig 3:**
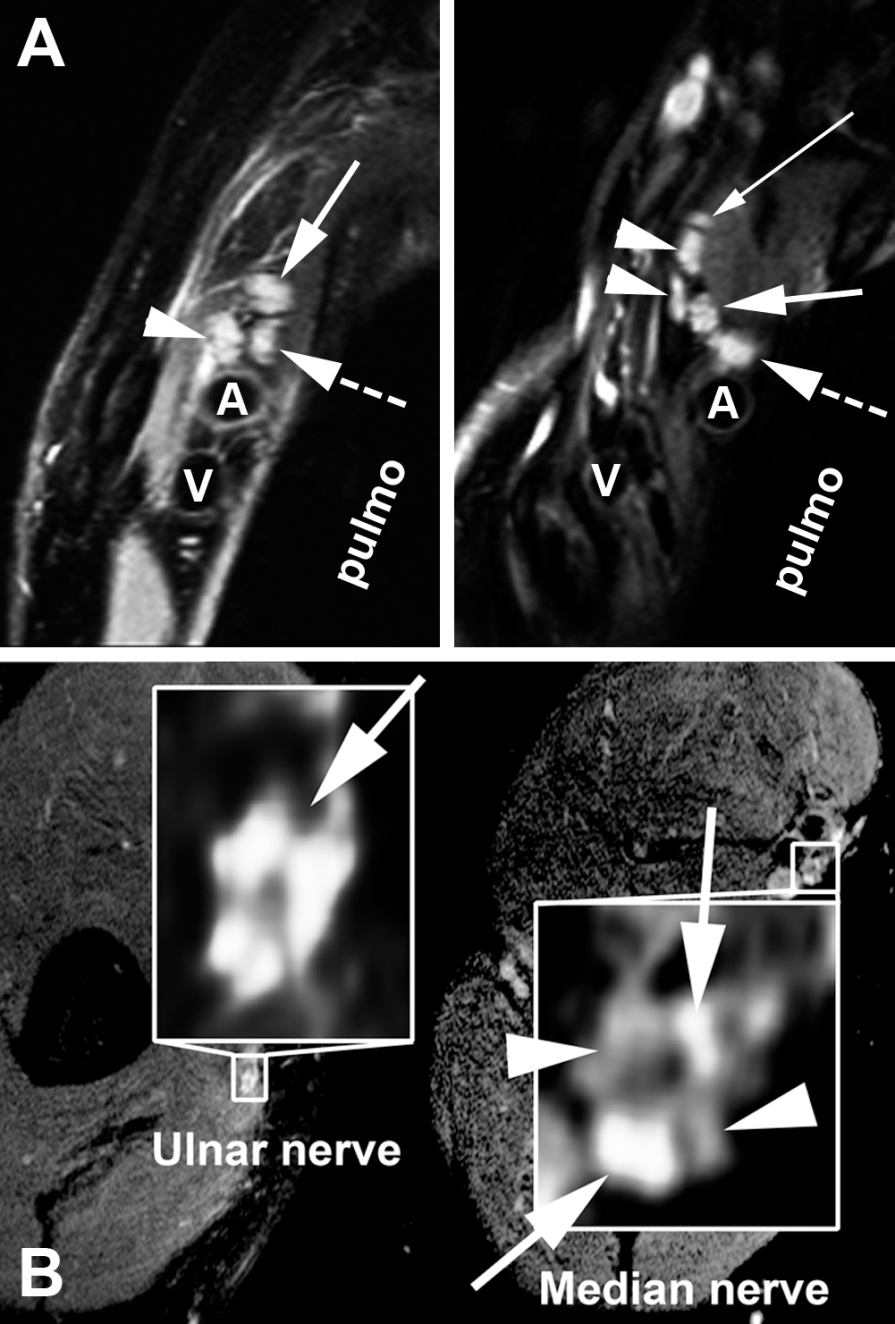
High-resolution magnetic resonance neurography in meningoencephaloradiculitis. HrMRN in plexus localisation (A): Pathological signal hyperintensities in high-resolution, T2-weighted coronal images of the brachial plexus. Left: The same patient as in Fig 2E and 2F. A representative section with perpendicular orientation to the lateral cord (arrowhead), medial cord (dashed arrow) and posterior cord is shown. The cords display strongly increased T2 signal and are swollen, indicative of a clinically relevant injury (high-grade flaccid tetraparesis, areflexia, no sensory deficit). Right: A 69-year-old female patient with high-grade flaccid paresis of the left arm. A representative section through the supraclavicular plexus at trunk level is shown, with strong T2 signal increase and calibre gain. The thin solid white arrow indicates the dorsal scapular nerve. The two arrowheads show the anterior and posterior divisions of the superior trunk; the thick solid white arrow, the middle trunk; the thick dashed white arrow, the inferior trunk. In both images, the letters A and V denote the subclavian artery and vein, respectively. "Pulmo" denotes the lung apex. hrMRN for peripheral nerve lesion localisation (B): Pathological signal hyperintensities in high-resolution, T2-weighted, axial images at upper arm level in a 62-year-old male patient suffering from flaccid paresis of the right arm. Left: A representative magnified section of the ulnar nerve, with a strong T2 lesion of the ulnar nerve fascicles (solid white arrow on left). Right: A zoomed, representative section of the median nerve of the same patient. The median nerve, in contrast to the ulnar nerve, shows a T2 lesion of some, but not all fascicles of its cross section. The arrowheads on the right indicate fascicles with normal/hypointense T2 signal, whereas the solid white arrows on the right designate lesion fascicles with clearly increased T2 signal. This imaging finding is in close agreement with the clinical symptoms of predominant ulnar neuropathy. Spatial resolution was 0.3–0.6 × 0.3–0.6mm in-plane at a slice thickness of 3–3.5mm.

Investigating AMNI in more detail, hrMRN was performed in patients with clinically suspected MER. In all investigated cases, lesion contrast was strong, with a pathologically hyperintense T2 signal of either nerve roots, plexus trunks, plexus fascicles or peripheral nerves. Furthermore, the calibre of affected neural segments was clearly enlarged. In most cases, the extent of spatial lesions and lesion patterns in hrMRN exceeded the clinical manifestations of disease. Fig 3A and 3B illustrates some examples of hrMRN pathology.

## Discussion

Overall, the distribution of the clinical manifestations in our cohort (pure meningitis 57.7%, ME 27.9% and MER 14.4%) is comparable with recent reports [11, 16, 17], although the proportion of MER was higher (14.4% versus 7.6–10%) and that of ME lower (27.9% versus 41–51.3%). Similarly, mortality was higher in our cohort (3.6% versus 0–1.5%)[11, 17, 18] and all those who died had presented with MER. The reasons remain unclear: mutations in TBEV causing more virulent strains have been discussed and, indeed, those strains were isolated in regions where uncommon, frequently severe cases developed [19]. Indicative of virus strain-dependent virulence and relation to severity of brain infection might be the fact that severe cases in risk areas in Europe range in frequency between 7.6% (e.g. Poland) and 14.1% (e.g. Latvia)[11, 16, 17, 20, 21]. Similarly, mortality in different risk areas in Europe ranges from 0% in Croatia, to 0.6% in Poland, 0.8% in the Czech Republic/Lithuania, and in Germany, respectively, 1.5% and 3.6%, as found in our study [11, 17, 18, 21, 22].

Recent studies have shown several clinical findings to be associated with severity of symptoms or late sequelae in TBE [11, 17, 21]. In our cohort, significantly more MER than ME patients presented with a consciousness disturbance at admission or which developed during the acute course (87.5% versus 45.2%, p = 0.003) (Fig 1A). In contrast, significantly more cerebellar symptoms such as ataxia (54.8%, p < 0.001) and oculomotor disturbances (29%, p = 0.018) were observed in ME. These data are partially consistent with previous clinical findings [11, 22–24]. However, there are several differences which may in part be due to our stringent classification into MER and ME. Although not statistically significant, higher-order brain dysfunction (such as aphasia) and autonomic symptoms were found exclusively in the ME group.

Taking the clinical features of MER and ME, respectively, together with a travel history from an endemic risk area, the evidence of peripheral flaccid paresis or prominent cerebellar signs should prompt the physician to suspect TBE. In cases of pure meningitis, a travel history from an endemic risk area and a history of tick-bite may support the suspected diagnosis but it cannot be clinically distinguished from other viral entities. In all cases, final diagnosis must be confirmed by the presence of TBEV antibodies (for details, see materials and methods, CSF analysis and immunoassays). If more complex symptoms such as delirious state, confusion, seizure or aphasia are present, other pathogens, especially members of the *herpesviridae*, should be taken into account [8, 10].

TBE not only has a potentially severe acute course but also produces long-term disability in a considerable number of individuals [21–24]. The mRS is a commonly used instrument to measure the degree of neurological disability affecting activities of daily living (ADL)[12]. Already at admission, the mRS score in MER and ME differed significantly (median 5 in MER versus 4 in ME, p < 0.001) (Table 2). At follow-up, the gap between MER and ME had widened, with ongoing severe disability in MER (median mRS 4 in MER versus 1 in ME, p < 0.0001). Looking more closely at the changes in mRS at follow-up (Fig 1B), all of the ME patients had mRS scores equal to or lower (“better”) than 3. In contrast, only 37.6% of MER patients scored equal to or lower (“better”) than 3. Another 37.5% were moderately severely disabled (mRS 4). Furthermore, 25% of MER and 3.6% of all patients died during intensive care or within the following month due to TBE or related complications (e.g. aspiration pneumonia). The significantly greater severity of the acute course in MER patients as well as the worse long-term outcome in our cohort are reflected in the significantly more frequent need for intensive care (100%, p < 0.0001) and mechanical ventilation (50%, p < 0.001) in the MER group during the acute infection phase (Table 2). Previous publications described rates of 36.5–43.3% moderate TBE and 8.2–12.7% severe TBE[18, 21–23]. In contrast to prior studies, we specifically addressed the clinical presentation of TBE with a stringent classification into MER and ME and the use of clinical outcome scales. Furthermore, as already discussed above for mortality, differences in virulence may account for the regional variability in severe courses.

We analysed putative risk factors and co-morbidity that might be associated with MER and a worse outcome. Higher age and male gender were identified as risk factors associated with both MER and ME. Thus, significantly more males were affected by MER (81.2%, p = 0.01); more males also presented with ME (71%, p = 0.027) than with meningitis (54.7%). Higher age was significantly associated with MER (median = 62 years, p<0.001) but the median age was also significantly higher in the ME group (57 years, p = 0.009) than for meningitis (46 years). Gender and age were both confirmed by logistic regression analyses as independent risk factors, especially for development of MER (Table 3). This is consistent with previous findings showing an increase in the relative frequency of severe TBE with increasing age [11, 18]. Moreover, using ordered logistic regression, we could show that every additional year (ten years) of age results in a 5% (50%) higher risk to switch from category meningitis to ME and from ME to MER, respectively (Table 3).

We collected further data to address the question of whether co-morbidity might be associated with severe course and alpha-motor neuron injury. We found that DM (43.8%, p = 0.024) and PNP (25%, p = 0.008) were observed significantly more frequently in the MER group than in the ME patients (Table 2). Logistic regression analysis showed that DM is an independent risk factor. Diabetic patients are regarded as immunocompromised and have a higher risk for infections in general [25, 26]. Interestingly, TBEV does not seem to be the only member of the *Flaviviridae* that is associated with diabetes. In addition to hepatitis C virus, most interestingly, DM has also been identified as a risk factor for West Nile virus (WNV) infections [27–29]. The latter is also a vector-borne infection with a clinical presentation quite similar to that of TBEV [4]. Furthermore, the WNV genome shares a high sequence homology with TBEV (approx. 54% sequence identity; sequence comparison with ALIGN, Optimal Global Sequence Alignment, e.g. Biology Workbench, http://workbenchsdsc.edu). One clue may be the fact that the receptors for virus cell entry, among others, are glycosaminoglycans that might be target proteins of non-enzymatic glycosylation on peripheral motor neurons in diabetics and thus may increase susceptibility to peripheral motor neuron infection [30].

MRI brain abnormalities in TBE have been reported in several previous studies. Correspondingly, we identified MRI abnormalities in 13% of the ME group and in 58% of the MER group (p = 0.005). In ME, all MRI-positive patients had thalamus lesions, and one patient also had evidence of pan encephalitis (Fig 2A and 2B). This is in accordance with previous findings that the thalamus seems to be a site of predilection [11, 31–34]. In some reports, mostly single cases or small case series, the extent of brain injury has been associated with disease severity [11, 31, 34, 35]. However, in these reports severity was defined by clinical presentation at admission, but the duration of follow-up was short or no follow-up was available. Only one comprehensive study conducted by Kaiser et al. showed a positive correlation of MRI abnormalities with severity of TBE [11], but they examined sequelae in general rather than differentiating between ME and MER. In contrast, we found a significant association of MRI findings with MER phenotype and worse long-term outcome (Table 2).

Using hrMRN, we found strong signal hyperintense lesion patterns of peripheral limb nerves in all examined MER patient’s. Here, hrMRN serves as a powerful diagnostic tool that not only can morphologically identify infection of peripheral nerves in general but can also give an exact neurotopic description of AMNI. HrMRN demonstrates fascicular lesions with strict somatotopic organisation and thus has an advantage over clinical and electrophysiological examinations (Fig 3A and 3B)[14, 15]. In this way hrMRN contributes substantially to estimating the prognosis of an individual patient.

## Conclusions

We have presented a comprehensive analysis of TBE from a high-risk area in south-western Germany. Our study does have a certain weakness in terms of power to compare MER cases. While we had a large initial sample size of 111 cases, breaking them down into clinical presentation categories and then examining differences between them weakened our ability to perform robust statistical analyses. This problem can only be solved by continuing to increase the sample size of cases prospectively and include participants from several European countries endemic for TBEV or by establishing a Europe-wide registry, both with consistent protocols. Nevertheless, our data should support neurologists and treating physicians from other specialties alike not only in endemic areas but, in particular, for treating travellers returning home from endemic areas to classify TBE and to estimate infection-associated complications and individual prognosis. In this context, hrMRN is a powerful tool to precisely describe peripheral motor neuron involvement in TBE-MER and thus has clinically relevant diagnostic implications for lesion localisation. Furthermore, our results document the increasing risk of severe, disabling infections in the elderly—especially in those individuals with a common and frequently observed comorbidity (such as diabetes)–and should prompt general practitioners in endemic countries to recommend vaccination for prevention of disease. Vaccine should also be recommended for those travelling to endemic areas, particularly those individuals who are older and expect to engage in activities that can increase their risk of exposure to ticks.

## Supporting Information

S1 Appendix Late sequelae in pure meningitis. (DOC)

S2 Appendix Epidemiological and clinical raw data. (XLSX)

## Abbreviations

AMNI: alpha-motor neuron injury
ME: meningoencephalitis
MER: meningoencephaloradiculitis
hrMRN: high-resolution magnetic resonance neurography
mRS: modified RANKIN scale
PNP: polyneuropathy
RKI: Robert Koch Institute Berlin, Germany [national infection surveillance centre]
SEM: standard error of mean
TBE: tick-borne encephalitis
TBEV: tick-borne encephalitis virus
