# Supplementary material for: Predictors, Neuroimaging Characteristics and Long-Term Outcome of Severe European Tick-Borne Encephalitis: A Prospective Cohort Study
Source: PLoS One. 2016 Apr 25;11(4):e0154143. doi: 10.1371/journal.pone.0154143 (PMC4844156; doi:10.1371/journal.pone.0154143)
Supplement: S1 Appendix — (DOC) [file pone.0154143.s001.doc]

**Late sequelae in pure meningitis patients.**

| Age, median (min.-max.) | **Meningitis^#^**  n = 59  46 (17–70) | | | |
| --- | --- | --- | --- | --- |
| ***Parameters*** | ***All patients*** | ***Male*** | ***Female*** | p |
| Any complaints | 20 (34%) | 9 (21%) | 11 (44%) | 0.129 |
| Dizziness | 6 (10%) | 1 (3%) | **5 (20%)** | **0.041** |
| Headache | 10 (17%) | 3 (9%) | 7 (28%) | 0.051 |
| Sleep disturbance | 5 (9%) | 3 (9%) | 2 (8%) | 0.7 |
| Neurasthenia | 11 (19%) | 6 (18%) | 5 (20%) | 0.517 |
| Depression | 2 (3%) | 1 (3%) | 1 (4%) | 0.663 |
| Others | 11 (19%) | 3 (9%) | 6 (24%) | 0.1 |
| mRS, median (min.-max.) | 0 (0-3) | 0 (0-3) | 0 (0-2) | - |
| Impairment of ADL* | 12 (57%) | 2 (25%) | **9 (75%)** | **0.04** |
| Reduced earning capacity | 3 (5%) | 1 (3%) | 2 (8%) | 0.374 |

^#^ w/o ME and MER; mRS, modified RANKIN-scale; ADL, activities of daily living; *of all patients that complained at least about one symptom; statistic was calculated with Fisher exact test.
